# Epidemiology and Outcomes of Non–Small Cell Lung Cancer in South Korea

**DOI:** 10.1001/jamanetworkopen.2023.55331

**Published:** 2024-02-09

**Authors:** Hyun Ae Jung, Dae Ho Lee, Sun Min Lim, Hyeyeon Yu, Shinkyo Yoon, DongKyu Kim, Kyu-pyo Kim, Hyehyun Jeong, Haewon Doh, Subin Lim, Joohyun Kim, Xiahong Zhao, David Horsburgh, Dony Patel, Jung-Ae Kim, Kong Chian Toh

**Affiliations:** 1 Division of Hematology-Oncology, Department of Medicine, Samsung Medical Center, Sungkyunkwan University School of Medicine, Seoul, South Korea; 2 Department of Oncology, Asan Medical Center, University of Ulsan College of Medicine, Seoul, South Korea; 3 Division of Medical Oncology, Department of Internal Medicine, Yonsei Cancer Center, Yonsei University College of Medicine, Seoul, South Korea; 4 Real-World Evidence Team, ALYND, Yonsei University Health System, Seoul, South Korea; 5 Department of Family Medicine, Yonsei University College of Medicine, Seoul, South Korea; 6 IQVIA Solutions, Real World Solutions–Korea, Seoul, South Korea; 7 IQVIA Solutions, Real World Solutions–Asia Pacific, Singapore; 8 IQVIA Solutions, Real World Solutions–Global, London, United Kingdom

## Abstract

**Importance:**

Valuable evidence regarding clinical characteristics, treatments, and outcomes for non–small cell lung cancer (NSCLC) is limited to individual hospital databases or national-level registries. The common data and federated analysis framework developed through the Extensible Platform for Observational Research in Lung Cancer (EXPLORE-LC) initiative allows for research across multiple high-quality data sources, which may provide a deeper understanding of the NSCLC landscape and identification of unmet needs of subpopulations.

**Objective:**

To describe clinical characteristics, initial treatment patterns, subsequent treatment, and overall survival (OS) of patients with NSCLC in South Korea.

**Design, Setting, and Participants:**

This multicenter cohort study included patients aged 18 years and older who were diagnosed with NSCLC between 2014 and 2019 and followed up until March 2020 at 3 tertiary hospitals in South Korea. Clinical data were collected using a common data model and clinical data warehouse. Patients who had an initial diagnosis of nonsquamous (NSQ) or squamous (SQ) NSCLC and who had received at least 1 treatment for NSCLC were included in the study. Data were analyzed from June through November 2022.

**Main Outcomes and Measures:**

The primary outcome was clinical OS for patients with NSCLC. Secondary outcomes were clinical characteristics and treatment patterns subsequent to the diagnosis of NSCLC.

**Results:**

Among 22 101 patients with NSCLC who received anticancer treatment analyzed in this study, 17 350 patients (78.5%) had NSQ and 4751 patients (21.5%) had SQ NSCLC. Clinical characteristics and outcomes and treatment patterns were assessed for 13 084 patients with NSQ cancer who had known *EGFR* and *ALK* status (75.4%; mean [SD] 62.2 [10.5] years; 6552 males [50.1%]) and all 4751 patients with SQ cancer (mean [SD] age, 67.1 [8.6] years; 4427 males [93.2%]). More than half of patients with NSQ cancer were never smokers (7399 patients [56.6%]). Patients with SQ cancer were mostly males and former or current smokers (4235 patients [89.1%]) and were diagnosed at a later clinical stage than patients with NSQ cancer (eg, stage I: 1165 patients [24.5%] vs 5388 patients [41.2%]). Patients with *EGFR*-positive and *ALK*-positive NSQ cancer diagnosed between 2017 and 2019 had better median OS than similar patients diagnosed between 2014 and 2016 (*EGFR*-positive: not reached [95% CI, 35.9 months to not reached] vs 28.4 months [95% CI, 25.8 to 30.0 months]; *P* < .001; *ALK*-positive: not reached [95% CI, not reached] vs 49.5 months [95% CI, 35.1 months to not reached]; *P* < .001). No significant difference was observed in OS from first-line treatment for patients with SQ cancer.

**Conclusions and Relevance:**

This study, which pooled medical data from multiple clinical data warehouses to produce a large study cohort, may provide meaningful insights into the clinical practice of NSCLC and underscores the value of a common data model approach. The analyzable dataset may hold great promise for future comprehensive identification of subpopulations and unmet needs.

## Key Points

**Question:**

Has overall survival (OS) of patients with non–small cell lung cancer (NSCLC) improved in recent years in clinical practice?

**Findings:**

This cohort study of 22 101 patients with NSCLC found that increased targeted therapy use for patients with nonsquamous cancer with *EGFR* sequence variants or *ALK* rearrangement in period 2 (2017-2019) was associated with better OS than for similar patients in period 1 (2014-2016). No significant difference in OS was observed between the periods for patients with squamous cancer.

**Meaning:**

This study used a common data model to collect and analyze medical data from clinical data warehouses in South Korea, providing insights into clinical management and outcomes of NSCLC and laying the groundwork for future analyses.

## Teaser

This cohort study investigates overall survival, along with clinical characteristics and treatment patterns, for patients with non–small cell lung cancer from 2014 to 2019 in South Korea.

## Introduction

Lung cancer is the leading cause of cancer deaths worldwide, with an estimated 2.2 million new diagnoses and 1.8 million deaths in 2020.^1^ Similar trends have been observed in South Korea, where there were 28 651 new diagnoses in 2020, with a 5-year, age-standardized prevalence of 92 diagnoses per 100 000 individuals.^2^

Non–small cell lung cancer (NSCLC) is characterized by a multitude of genetic alterations that drive oncogenesis. These biomarkers are excellent targets, and treatments developed for these targets have shown the ability to improve the survival of patients with advanced NSCLC (aNSCLC) by 15% to 50%.^3,4,5^ Current National Comprehensive Cancer Network guidelines suggest molecular testing for the following 8 druggable biomarkers in aNSCLC: anaplastic lymphoma kinase (*ALK*) rearrangements, B-Raf proto-oncogene (*BRAF* V600E), epidermal growth factor receptor (*EGFR*), Kirsten rat sarcoma virus (*KRAS* G12C), mesenchymal-epithelial transition (*MET*) exon 14 skipping sequence variants, neurotrophic receptor tyrosine kinase (*NTRK1/2/3*) fusions, rearranged during transfection (*RET*) rearrangements, and c-ros oncogene 1 (*ROS1*) rearrangement, in addition to programmed cell death ligand 1 (PDL-1) expression levels.^6^

This area of research has progressed rapidly, with discoveries of new driver sequence variations and the development of targeted therapies and combination treatment strategies. As a result, the journey of patients with NSCLC is diverse and undergoes multiple processes and overall treatment durations have been extended. Information collected as part of routine medical care and stored in electronic databases has potential as a reliable data source for generating clinical evidence. However, differences in data structure across hospitals mean that multisite research on the treatment landscape and clinical outcomes for aNSCLC and biomarker subpopulations is lacking in South Korea. To address this need, the Extensible Platform for Observational Research in Lung Cancer (EXPLORE-LC) was initiated as a multinational, multisite collaboration to develop a common data standard and federated network in support of clinical evidence generation in NSCLC across the Asia-Pacific region.^7,8^ This combined analysis provides one of the largest NSCLC research networks, offering a unique approach to understand changing treatment patterns and clinical outcomes specific to the Asia-Pacific region.

This study describes patient characteristics, treatment patterns, and outcomes of NSCLC in South Korea. Study findings may improve understanding of the treatment of patients with NSCLC and provide a platform to address evidence gaps between clinical research and clinical practice.

## Methods

This cohort study follows the Strengthening the Reporting of Observational Studies in Epidemiology (STROBE) reporting guideline for cohort studies and was approved by each site’s institutional review board (IRB): the Samsung Medical Center IRB, Asan Medical Center IRB, and Severance Hospital IRB. The need for informed consent was waived by each institution’s IRB owing to the retrospective nature of this study.

### Patients, Study Design, and Data Collection

This multicenter, retrospective cohort study analyzed clinical data warehouses from 3 large tertiary hospitals (Samsung Medical Center, Asan Medical Center, and Severance Hospital). Together, the data included represent approximately 20% to 25% of all NSCLC diagnoses between 2014 and 2019 in South Korea.^9,10^ Data were extracted for patients who met the following inclusion criteria: aged 18 years or older at initial diagnosis of NSCLC between January 2014 and December 2019 and received at least 1 treatment for NSCLC during the study period at the participating hospital. Patients with a history of previous or concurrent primary cancer except NSCLC, diagnosed during or up to 6 months before the diagnosis of NSCLC, or with histopathological *International Classification of Diseases for Oncology, Third Revision* (*ICD-O-3*) codes indicating SCLC were excluded. See eTable 1 in Supplement 1 for *International Statistical Classification of Diseases and Related Health Problems, Tenth Revision* (*ICD-10*) codes used to define previous or concurrent primary cancer and *ICD-O-3* codes used to define histopathologically confirmed NSCLC.

Baseline demographic and clinical characteristics (age, sex, smoking status, performance status, American Joint Committee on Cancer tumor, node, and metastasis clinical stage, and histology) were extracted. Structured data (any predefined, formatted, and coded data) were extracted from clinical data warehouses. Enhancement via manual abstraction or text-mining was performed for unavailable or unstructured key variables (from written medical records). Biomarker testing information from routine clinical practice was also collated from polymerase chain reaction, fluorescence in situ hybridization, immunohistochemistry, and next-generation sequencing (NGS) reports, which were then structured and standardized using a combination of text-mining and manual abstraction.

Variables that are not often directly captured, such as line of therapy (LoT) and multimodal treatment plans, were derived using treatment type and dates. Physicians from the 3 hospitals agreed to the algorithm used to derive LoT. Initial treatment was defined as the first treatment received within 6 months of diagnosis, and advancement to the next treatment line was based on the switching of individual agents within regimens and time between defined treatment regimens.

All data were extracted and prepared according to a data specification to ensure a consistent data definition. Subsequently, all extracted data were transformed into the EXPLORE-LC common data model. All data collected were dependent on data availability, quality, and validity, which were evaluated through data due diligence prior to analysis to ensure data conformity, completeness, and plausibility.

### Outcomes and Measurements

The primary outcomes were OS from diagnosis and OS from first LoT. These were found for patients with NSQ NSCLC stratified by *EGFR* and *ALK* status and separately for patients with SQ NSCLC stratified by the period of diagnosis (period 1 [2014-2016] and period 2 [2017-2019]).

Secondary outcomes included baseline demographics and clinical characteristics. These were found for patients with NSQ NSCLC stratified by *EGFR* and *ALK* status and separately for patients with SQ NSCLC stratified by period 1 and period 2. Initial treatments of patients with NSQ and SQ NSCLC stratified by stage IIIA and stage IIIB/C and by period 1 and period 2 were also included. Treatment patterns of systemic anticancer therapy (SACT)–treated aNSCLC from first to third LoT were compared between period 1 and period 2.

### EXPLORE-LC Data

EXPLORE-LC uses a 2-stage data analysis approach following the common data model method.^11,12^ First, site research teams analyzed deidentified, patient-level data on site and generated aggregated statistical summaries in a common format and representation.^11,12^ Second, aggregated statistical summaries of study variables of interest and analyses were shared to a central location and pooled using meta-analysis. No patient-level data were transmitted outside of the hospital for analysis. This 2-stage strategy was created to address data privacy issues in South Korea while also facilitating multisite research.

### Statistical Analysis

Categorical variables included patient characteristics, such as biomarker status, and initial treatments; these were described as counts and percentages. Continuous variables were summarized using means with SDs and medians with IQRs wherever appropriate. The comparison of PDL-1 levels between patients with and without any potentially major druggable sequence variations was conducted using the Fisher exact test. Palliative treatment sequences of patients initially diagnosed with aNSCLC were displayed using a Sankey diagram for the first 3 LoTs. We analyzed OS using the Kaplan Meier approach, including median survival estimates with 95% CIs and 2-sided *P* values from log-rank tests. All statistical analyses were performed using R statistical software version 4.1.1 (R Project for Statistical Computing). Statistical significance was assessed at a level of P < .05. Data were analyzed from June through November 2022.

The number and percentage of missing data was reported, and no imputation was made for missing data in the original data source. This was a descriptive study with no a priori hypothesis testing; therefore, no formal calculation of sample size or statistical power was required.

## Results

A total of 22 101 patients with NSCLC who received anticancer treatment between 2014 and 2019 were included in this study, including 17 350 patients (78.5%) with NSQ and 4751 patients (21.5%) with SQ histology. Clinical characteristics and outcomes and treatment patterns were assessed for 13 084 patients with NSQ cancer (75.4%; mean [SD] 62.2 [10.5] years; 6552 males [50.1%]) who were *EGFR* wild type and *ALK* wild type (WT subgroup) or had *EGFR* sequence variation (*EGFR*-positive subgroup) or *ALK* rearrangement (*ALK*-positive subgroup) and all 4751 patients with SQ cancer (mean [SD] age, 67.1 [8.6] years; 4427 males [93.2%]).

### Baseline Characteristics of Patients with NSQ Cancer by *EGFR* and *ALK* Status

Among patients with NSQ cancer and known *EGFR* and *ALK* status, 5304 individuals (40.5%) were WT, 6866 individuals (52.5%) were *EGFR*-positive, and 914 individuals (7.0%) were *ALK*-positive. The median (IQR) age of all patients with NSQ was 63 (56- 70) years. Patients with *ALK*-positive cancer (median [IQR] age, 55 [47-67] years) were younger than patients with *EGFR*-positive (median [IQR] age, 62 [56-69] years) and WT (median [IQR] age, 64 [57-71] years) cancer. There was a higher proportion of females in the *EGFR*-positive (4310 females [62.8%]) and *ALK*-positive (525 females [57.4%]) subgroups than the WT subgroup (1697 females [32.0%]). More than half of patients with NSQ cancer (7399 patients [56.6%]) were never smokers. The proportion of never smokers was higher in *EGFR*-positive (4763 patients [69.4%]) and *ALK*-positive (594 patients [65.0%]) subgroups than the WT subgroup (2042 patients [38.5%]). At the time of initial NSCLC diagnosis, 5388 patients (41.2%), 1056 patients (8.1%), 1654 patients (12.6%), and 4986 patients (38.1%) were diagnosed at clinical stage I, II, III, and IV, respectively (Table 1).

**Table 1.** Characteristics of Patients With NSQ NSCLC

Characteristic at initial diagnosis	Patients, No. (%)^a^			
	All (n = 13 084)^a^	*EGFR* WT and *ALK* WT (n = 5304)	*EGFR* variant (n = 6866)^b^^,^^c^	*ALK* variant (n = 914)^b^^,^^d^
Diagnosis year				
Period 1 (2014-2016)	5859 (44.8)	2477 (46.7)	2965 (43.2)	417 (45.6)
Period 2 (2017-2019)	7225 (55.2)	2827 (53.3)	3901 (56.8)	497 (54.4)
Age, y				
Mean (SD)	62.2 (10.5)	63.6 (10.0)	62.1 (10.3)	55.3 (12.0)
Median (IQR)	63.0 (56.0-70.0)	64.0 (57.0-71.0)	62.0 (56.0-69.0)	55.0 (47.0-64.0)
Range	19.0-94.0	20.0-92.0	19.0-94.0	19.0-88.0
Sex				
Female	6532 (49.9)	1697 (32.0)	4310 (62.8)	525 (57.4)
Male	6552 (50.1)	3607 (68.0)	2556 (37.2)	389 (42.6)
Smoking status				
Never smoker	7399 (56.6)	2042 (38.5)	4763 (69.4)	594 (65.0)
Former smoker	3641 (27.8)	1955 (36.9)	1489 (21.7)	197 (21.6)
Current smoker	2019 (15.4)	1297 (24.5)	600 (8.7)	122 (13.4)
Missing or unknown	25 (0.2)	10 (0.2)	14 (0.2)	<3 (<0.3)
ECOG performance status score				
0	4943 (37.8)	1960 (37.0)	2716 (39.6)	267 (29.2)
1	3799 (29.0)	1620 (30.5)	1839 (26.8)	340 (37.2)
2	318 (2.4)	156 (2.9)	139 (2.0)	23 (2.5)
3	28 (0.2)	10 (0.2)	16 (0.2)	<3 (<0.3)
4	8 (0.1)	<3 (<0.1)	6 (0.1)	0
Missing or unknown	3988 (30.5)	1556 (29.3)	2150 (31.3)	282 (30.9)
TNM classification				
IA	3962 (30.3)	1466 (27.6)	2327 (33.9)	169 (18.5)
IB	1426 (10.9)	554 (10.4)	810 (11.8)	62 (6.8)
IIA	514 (3.9)	234 (4.4)	242 (3.5)	38 (4.2)
IIB	542 (4.1)	293 (5.5)	220 (3.2)	29 (3.2)
IIIA	1113 (8.5)	549 (10.4)	461 (6.7)	103 (11.3)
IIIB/C	541 (4.1)	311 (5.9)	173 (2.5)	57 (6.2)
IV	4986 (38.1)	1897 (35.8)	2633 (38.4)	456 (49.9)
Biomarker test result				
PDL-1 expression				
Not tested	6982 (53.4)	2700 (50.9)	3756 (54.7)	526 (57.6)
Tested but no valid result	131 (1.0)	80 (1.5)	43 (0.6)	8 (0.9)
<1%	3082 (23.6)	1189 (22.4)	1777 (25.9)	116 (12.7)
1% to <50%	1592 (12.2)	648 (12.2)	825 (12.0)	119 (13.0)
≥50%	1292 (9.9)	683 (12.9)	464 (6.8)	145 (15.9)
Missing or unknown	5 (0.1)	4 (0.1)	<3 (<0.1)	0

Abbreviations: *ALK*, anaplastic lymphoma kinase; ECOG, Eastern Cooperative Oncology Group; *EGFR*, epidermal growth factor receptor; NSCLC, non–small cell lung cancer; NSQ, nonsquamous; PDL-1, programmed cell death ligand 1; TNM, tumor, node, metastasis; WT, wild type.

^a^ Includes *EGFR* variant, *ALK* variant, and *EGFR* and *ALK* WT subtypes and excludes other variant subtypes and co-occurring sequence variations.

^b^ Variant is used interchangeably and can refer to a sequence variation, alteration, or (over)expression depending on the molecular marker tested.

^c^ *EGFR* variants, defined as all *EGFR*-activating variants, include but are not limited to exon 19 deletion, exon 21 L858R substitution, and exon 20 insertion.

^d^ *ALK* variants are also known as *ALK* rearrangements or translocations.

### Baseline Characteristics of Patients With SQ Cancer

Among patients with SQ cancer, 2278 individuals (48.0%) were diagnosed in period 1 (2014-2016) and 2473 individuals (52.0%) were diagnosed in period 2 (2017-2019). There were no significant differences in clinical characteristics between patients diagnosed in period 1 and period 2. The median (IQR) age of all patients with SQ was 67 (62-73) years. Contrary to the NSQ group, more males (4427 males [93.2%]) than females (324 females [6.8%]) had SQ. Most patients with SQ cancer were former (2462 individuals [51.8%]) or current (1773 individuals [37.3%]) smokers (4235 patients [89.1%] total). The proportion of never smokers among patients with SQ cancer (510 patients [10.7%]) was much lower than among patients with NSQ cancer. At the time of initial NSCLC diagnosis, 1165 patients (24.5%), 978 patients (20.6%), 1530 patients (32.2%), and 1078 patients (22.7%) were diagnosed at clinical stage I, II, III, and IV, respectively (Table 2).

**Table 2.** Characteristics of Patients With SQ NSCLC

Characteristic at initial diagnosis	Patients, No. (%)		
	All (n = 4751)	Period 1 (n = 2278)^a^	Period 2 (n = 2473)^b^
Diagnosis year			
Period 1 (2014-2016)	2278 (48.0)	2278 (100)	0
Period 2 (2017-2019)	2473 (52.1)	0	2473 (100)
Age, y			
Mean (SD)	67.1 (8.6)	66.6 (8.7)	67.5 (8.5)
Median (IQR)	67.0 (62.0-73.0)	67.0 (61.0-73.0)	68.0 (62.0-74.0)
Range	26.0-93.0	26.0-93.0	29.0-91.0
Sex			
Female	324 (6.8)	147 (6.5)	177 (7.2)
Male	4427 (93.2)	2131 (93.6)	2296 (92.8)
Smoking status			
Never smoker	510 (10.7)	215 (9.4)	295 (11.9)
Former smoker	2462 (51.8)	1248 (54.8)	1214 (49.1)
Current smoker	1773 (37.3)	812 (35.7)	961 (38.9)
Missing or unknown	6 (0.1)	3 (0.1)	3 (0.1)
ECOG performance status score			
0	1353 (28.5)	430 (18.9)	923 (37.3)
1	1692 (35.6)	831 (36.5)	861 (34.8)
2	198 (4.2)	118 (5.2)	80 (3.2)
3	25 (0.5)	12 (0.5)	13 (0.5)
4	3 (0.1)	<3 (<0.1)	<3 (<0.1)
Missing or unknown	1480 (31.2)	886 (38.9)	594 (24.0)
TNM classification			
IA	571 (12.0)	287 (12.6)	284 (11.5)
IB	594 (12.5)	323 (14.2)	271 (11.0)
IIA	487 (10.3)	294 (12.9)	193 (7.8)
IIB	491 (10.3)	191 (8.4)	300 (12.1)
IIIA	987 (20.8)	491 (21.6)	496 (20.1)
IIIB/C	543 (11.4)	207 (9.1)	336 (13.6)
IV	1078 (22.7)	485 (21.3)	593 (24.0)
Biomarker test result			
*EGFR*^c^			
Not tested	2610 (54.9)	1365 (59.9)	1245 (50.3)
Tested but no valid result	<3 (<0.1)	0	<3 (<0.1)
WT	2045 (43.0)	881 (38.7)	1164 (47.1)
Exon 19 deletion sequence variation	36 (0.8)	13 (0.6)	23 (0.9)
Exon 21 L858R substitution sequence variation	21 (0.4)	9 (0.4)	12 (0.5)
Exon 20 insertion sequence variation	7 (0.2)	0	7 (0.3)
T790M sequence variation	8 (0.2)	5 (0.2)	3 (0.1)
Other *EGFR* sequence variation	23 (0.5)	5 (0.2)	18 (0.7)
Missing or unknown	0	0	0
* ALK*			
Not tested	2674 (56.3)	1395 (61.2)	1279 (51.7)
Tested but no valid result	<3 (<0.1)	0	<3 (<0.1)
WT	2059 (43.3)	878 (38.5)	1181 (47.8)
Sequence variation^d^	15 (0.3)	4 (0.2)	11 (0.4)
Missing or unknown	<3 (<0.1)	<3 (<0.1)	<3 (<0.1)
* ROS1*			
Not tested	4480 (94.3)	2241 (98.4)	2239 (90.5)
Tested but no valid result	<3 (<0.1)	0	<3 (<0.1)
WT	268 (5.6)	35 (1.5)	233 (9.4)
Sequence variation^e^	<3 (<0.1)	<3 (<0.1)	0
Missing or unknown	<3 (<0.1)	<3 (<0.1)	0
* BRAF*			
Not tested	4531 (95.4)	2249 (98.7)	2282 (92.3)
Tested but no valid result	0	0	0
WT	215 (4.5)	28 (1.2)	187 (7.6)
Sequence variation^e^	5 (0.1)	<3 (<0.1)	4 (0.2)
Missing or unknown	0	0	0
* RET*			
Not tested	4543 (95.6)	2250 (98.8)	2293 (92.7)
Tested but no valid result	0	0	0
WT	206 (4.3)	27 (1.2)	179 (7.2)
Sequence variation^e^	<3 (<0.1)	<3 (<0.1)	<3 (<0.1)
Missing or unknown	0	0	0
* KRAS*			
Not tested	4384 (92.3)	2098 (92.1)	2286 (92.4)
Tested but no valid result	0	0	0
WT	349 (7.4)	177 (7.8)	172 (7.0)
Sequence variation^e^	18 (0.4)	3 (0.1)	15 (0.6)
Missing or unknown	0	0	0
*MET*^f^			
Not tested	4537 (95.5)	2248 (98.7)	2289 (92.6)
Tested but no valid result	<3 (<0.1)	<3 (<0.1)	0
WT	190 (4.0)	25 (1.1)	165 (6.7)
*MET* exon 14 skipping	3 (0.1)	<3 (<0.1)	<3 (<0.1)
Amplification sequence variation	9 (0.2)	<3 (<0.1)	8 (0.3)
Other sequence variation	11 (0.2)	<3 (<0.1)	10 (0.4)
Missing or unknown	0	0	0
*ERBB2* (formerly *HER2*)			
Not tested	4540 (95.6)	2253 (98.9)	2287 (92.5)
Tested but no valid result	<3 (<0.1)	0	<3 (<0.1)
WT	201 (4.2)	22 (1.0)	179 (7.2)
Sequence variation^e^	9 (0.2)	3 (0.1)	6 (0.2)
Missing or unknown	0	0	0
PDL-1 expression			
Not tested	2777 (58.5)	1804 (79.2)	973 (39.3)
Tested but no valid result	46 (1.0)	43 (1.9)	3 (0.1)
<1%	518 (10.9)	144 (6.3)	374 (15.1)
≥1% and <50%	718 (15.1)	157 (6.9)	561 (22.7)
≥50%	691 (14.5)	130 (5.7)	561 (22.7)
Missing or unknown	<3 (<0.1)	0	<3 (<0.1)

Abbreviations: *ALK*, anaplastic lymphoma kinase; *BRAF*, B-Raf proto-oncogene; ECOG, Eastern Cooperative Oncology Group; *EGFR*, epidermal growth factor receptor; *ERBB2*, human epidermal growth factor receptor 2; *KRAS*, Kirsten rat sarcoma virus; *MET*, mesenchymal-epithelial transition; NSCLC, non–small cell lung cancer; PDL-1, programmed cell death ligand 1; *RET*, rearranged during transfection; *ROS1*, c-ros oncogene 1; SQ, squamous; TNM, tumor, node, metastasis; WT, wild type.

^a^ Period 1 was 2014 to 2016.

^b^ Period 2 was 2017 to 2019.

^c^ *EGFR* variants, defined as all *EGFR*-activating variants, include but are not limited to exon 19 deletion, exon 21 L858R substitution, and exon 20 insertion.

^d^ *ALK* variants are also known as *ALK* rearrangements or translocations.

^e^ Variant is used interchangeably and can refer to a sequence variation, alteration, or (over)expression depending on the molecular marker tested.

^f^ *MET* abnormalities include but are not limited to exon 14 skipping and amplification.

### Biomarker Status

**Figure 1.**
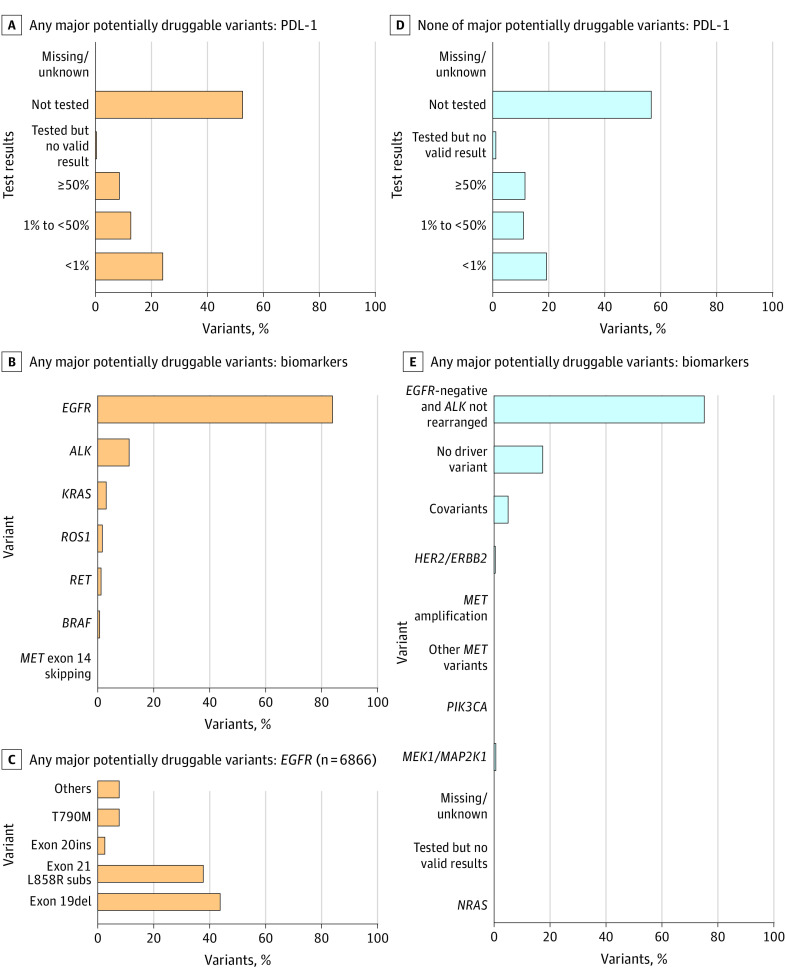
Biomarker Results by Druggable Variation Type for Patients With Nonsquamous Cancer PDL-1 indicates programmed cell death ligand 1.

Among patients with NSQ cancer, 8332 individuals (48.0%) had a single sequence variation and 391 patients (2.3%) had co-occurring sequence variations (Figure 1; eTable 2 in Supplement 1). Patients with NSQ vs SQ had a greater chance (8222 patients [47.4%] vs 109 patients [2.3%]) of detection of any potentially major druggable variant: *EGFR* variant (6866 patients [39.6%]), *ALK* rearrangement (914 patients [5.3%]), *KRAS* variant (239 patients [1.4%]), *ROS1* rearrangement (98 patients [0.6%]), *RET* alteration (59 patients [0.3%]), *BRAF* variant (32 patients [0.2%]), and *MET* exon 14 skipping (14 patients [0.1%]) (eTable 2 in Supplement 1). Among patients with *EGFR*-positive NSQ cancer, subtypes were exon-19 deletion (2999 patients [43.7%]), exon-21 L858R substitution (2591 patients [37.7%]), T790M (556 patients [8.1%]), exon-20 insertion (185 patients [2.7%]), and other *EGFR* variants (535 patients [7.8%]) (Figure 1C). Among 85 patients with *EGFR*-positive SQ cancer, subtypes were exon-19 deletion (33 patients [38.8%]), exon-21 L858R substitution (21 patients [24.7%]), T790M (8 patients [9.4%]), exon-20 insertion (7 patients 8.2%), and other *EGFR* variants (16 patients [18.8%]) (eFigure 1 in Supplement 1).

Among patients with NSQ cancer, there was a significant difference in PDL-1 expressors levels between 8222 patients with any potentially major druggable variants and 7070 patients without potentially major druggable variants (PDL-1 ≥50%: 718 patients [8.7%] vs 833 patients [11.8%]; PDL-1 1% to <50%: 1045 patients [12.7%] vs 767 patients [10.8%]; PDL-1 <1%: 1985 patients [24.1%] vs 1361 patients [19.2%]; *P* < .001) (Figure 1A and D). PDL-1 expressor levels for patients with NSQ cancer without any molecular testing are depicted in eFigure 2 in Supplement 1.

### Initial Treatment Patterns and Survival Outcomes of Stage IIIA and Stage IIIB/C

Among 2547 patients diagnosed with stage IIIA disease, surgery plus SACT and radiotherapy was the most common initial treatment in period 1 (398 of 1165 patients [34.2%]) and period 2 (422 of 1382patients [30.5%]). This was followed by surgery plus SACT as the second most common initial treatment in period 1 (186 patients [16.0%]) and period 2 (330 patients [23.9%]) (eFigure 3 in Supplement 1).

For 1330 patients with stage IIIB/C cancer, SACT plus radiotherapy (concurrent chemoradiation) was the predominant initial treatment option in period 1 (303 of 513 patients [59.1%]) and period 2 (453 of 817 patients [55.5%]), followed by SACT only as the second most common initial treatment in period 1 (113 patients [22.0%]) and period 2 (175 patients [21.4%]) (eFigure 3 in Supplement 1). OS outcomes from diagnosis according to initial treatment received are presented in eFigure 8 in Supplement 1.

### Treatment Sequence of aNSCLC by Year of Diagnosis and *EGFR* and *ALK* Status

Among 1149 patients with *EGFR*-positive NSQ aNSCLC diagnosed in period 1, 929 patients (80.9%) and 293 patients (25.5%) received targeted therapy (TKI) at first and second line, respectively. Of patients treated with first-line TKI, 169 patients (18.2%), 167 patients (18.0%), and 85 patients (9.1%) subsequently received TKIs, non–platinum-based chemotherapy alone, and platinum-based chemotherapy alone, respectively, as second-line treatment (eFigure 4 in Supplement 1). For 1412 patients with *EGFR*-positive NSQ aNSCLC diagnosed in period 2, 1142 patients (80.9%) and 379 patients (26.8%) received TKIs at first line and second line, respectively. Among patients treated with first-line TKIs, 265 patients (23.2%), 125 patients (10.9%), and 49 patients (4.3%) subsequently received TKIs, platinum-based chemotherapy alone, and non–platinum-based chemotherapy alone, respectively, as second-line treatment (eFigure 4 in Supplement 1).

Among 196 patients with *ALK*-positive NSQ aNSCLC diagnosed in period 1, 114 patients (58.2%) received platinum-based chemotherapy alone at first line and 113 patients (57.7%) received TKIs at second line. Of patients treated with platinum at first line, 84 patients (73.7%) subsequently received TKIs at second line, while 4 patients (18.2%) treated with first-line TKIs received other *ALK* TKIs at second line (eFigure 5 in Supplement 1). Among 277 patients with *ALK*-positive NSQ aNSCLC diagnosed in period 2, 192 patients (69.3%) and 118 patients (42.6%) received TKIs at first line and second line, respectively. Of patients treated with first-line TKIs, 74 patients (38.5%) subsequently received other *ALK* TKIs at second line (eFigure 5 in Supplement 1).

Among 883 patients with *EGFR* and *ALK* WT NSQ aNSCLC diagnosed in period 1, 693 patients (78.5%) received platinum-based chemotherapy alone at first line and 272 patients (30.8%) received non–platinum-based chemotherapy alone at second line. Of patients treated with first-line platinum, 243 patients (35.1%) subsequently received non–platinum-based chemotherapy alone at second line (eFigure 6 in Supplement 1). Among 995 patients with *EGFR* and *ALK* WT NSQ aNSCLC diagnosed in period 2, 717 patients (72.1%) received platinum-based chemotherapy alone at first line and 246 patients (24.7%) received checkpoint inhibitors (ICIs) at second line. Of patients treated with first-line platinum, 235 patients (32.8%) subsequently received ICIs at second line (eFigure 6 in Supplement 1).

Among 468 patients with SQ aNSCLC diagnosed in period 1, 362 patients (77.4%) received platinum-based chemotherapy alone at first line and 135 patients (28.8%) received non–platinum-based chemotherapy alone at second line. Of patients treated with first-line platinum, 113 patients (15.8%) subsequently received non–platinum-based chemotherapy alone at second line (eFigure 7 in Supplement 1). Among 701 patients with SQ aNSCLC diagnosed in period 2, 487 patients (69.5%) received platinum-based chemotherapy alone at first-line and 237 patients (33.8%) received ICIs at second line. Of patients treated with first-line platinum, 223 patients (45.8%) subsequently received ICIs at second line (eFigure 7 in Supplement 1).

### NSQ Overall Survival by *EGFR* and *ALK* Status

**Figure 2.**
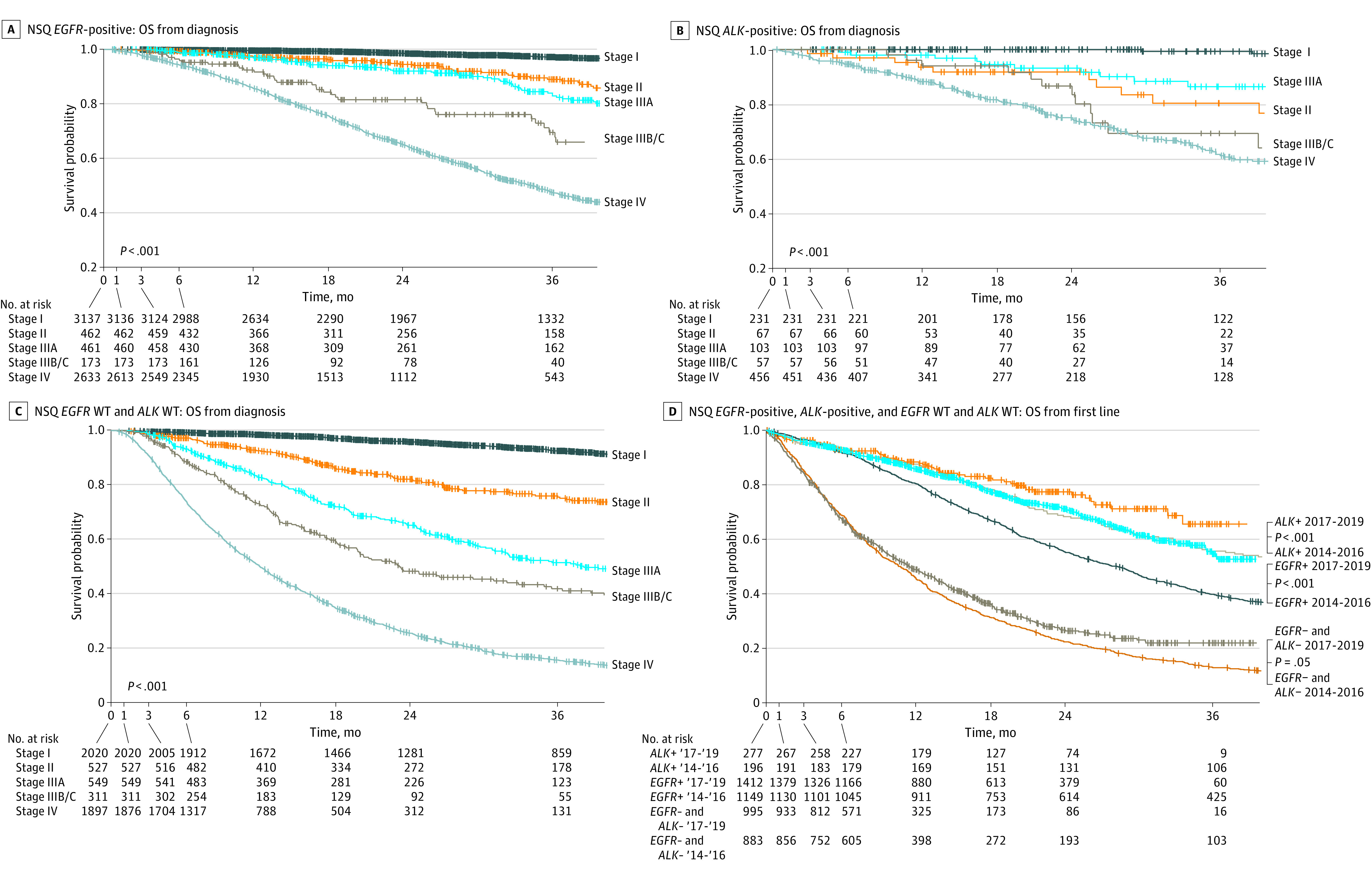
Overall Survival (OS) From Diagnosis by Clinical Stage in Nonsquamous (NSQ) Cancer OS is presented in NSQ non–small cell lung cancer among patients with (A) epidermal growth factor receptor (*EGFR*)–positive, (B) anaplastic lymphoma kinase (*ALK*)–positive, and (C) *EGFR* and *ALK* wild type (WT) cancer and (D) from first-line treatment by year of diagnosis.

Patients with stage IV NSQ cancer had the worst survival outcomes compared with other stages across *EGFR*-positive, *ALK*-positive, and WT subgroups (*P* < .001) (Figure 2A, B, and C). The median OS for stage IV NSQ cancer was 34.6 months (95% CI, 32.2-36.1 months), 56.4 months (95% CI, not reached), and 12.0 months (95% CI, 11.4-12.8 months), for *EGFR*-positive, *ALK*-positive, and WT subgroups, respectively (eTable 3 in Supplement 1).

Patients with *EGFR*-positive or *ALK*-positive NSQ aNSCLC in period 2 had better OS than patients from period 1 (*P* < .001) (Figure 2D). The median OS for these patients was not reached (95% CI, 35.9 months to not reached) for period 2 in *EGFR*-positive NSQ cancer and not reached (95% CI, not reached) in *ALK*-positive NSQ cancer and 28.4 months (95% CI, 25.8 to 30.0 months) in *EGFR*-positive NSQ cancer and 49.5 months (95% CI, 35.1 months to not reached) in *ALK*-positive NSQ cancer for period 1. A significant difference in OS was also observed in the WT subgroup across periods (*P* = .049) (Figure 2D). The median OS for these patients was 11.6 months (95% CI, 10.3 to 13.4 months) for period 2 and 10.8 months (95% CI, 9.6 to 11.9 months) for period 1.

### SQ Overall Survival by Stage and Year of Diagnosis

**Figure 3.**
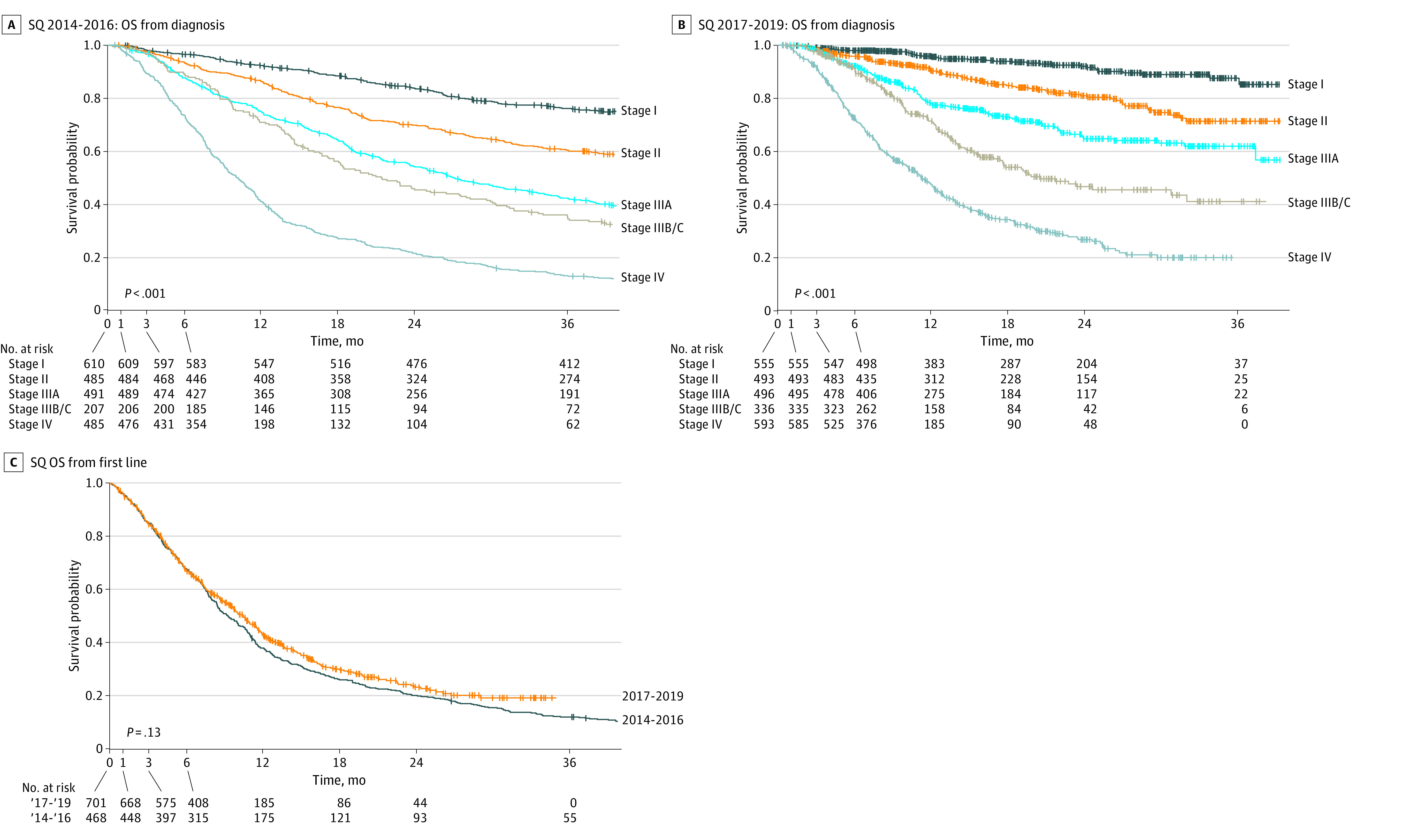
Overall Survival (OS) From Diagnosis by Clinical Stage in Squamous (SQ) Cancer OS is presented in SQ non–small cell lung cancer among patients diagnosed in (A) period 1 (2014-2016) and (B) period 2 (2017-2019) and (C) from first-line treatment.

Among patients with SQ cancer, significant differences in OS between clinical stages were observed (*P* < .001) across periods (Figure 3A and B). In period 1, the median OS for patients with SQ was 68.6 months (95% CI, not reached), 27.2 months (95% CI, 24.1-32.9 months), 21.5 months (95% CI, 17.7-28.1 months), and 10.2 months (95% CI, 9.2-11.3 months) for stage II, IIIA, IIIB/C, and IV, respectively (eTable 3 in Supplement 1). The median OS from first-line treatment was not reached for stage I. No significant difference was observed in OS from first-line treatment for patients with SQ aNSCLC across periods (Figure 3C).

## Discussion

This cohort study describes the epidemiology, treatment patterns, and outcomes of NSCLC in a clinical setting in South Korea across 6 years. It describes demographics, clinical characteristics, survival outcomes, and treatment patterns for patients with NSCLC by histology and *EGFR* and *ALK* status.

Results from 17 835 patients with NSCLC analyzed in this study were consistent with those of previous clinical studies from South Korea.^13,14,15^ The distribution of key characteristics observed were also in agreement with data obtained from the Korea Central Cancer Registry, in which most patients with NSCLC were diagnosed at stage IV, consistent with recent trends in the Korean population.^13,14,16^

In our study, more than half of patients with NSQ NSCLC had at least 1 potentially druggable sequence variation, and of these, *EGFR*-positive cancers were most frequent. Among patients with *EGFR*-positive NSQ cancer, exon-19 deletion variants occurred in almost half of patients, followed by exon-21 L858R substitution. Similar distribution of *EGFR* subtypes was previously seen in clinical studies from South Korea.^5^ Patients with SQ NSCLC were not generally tested for biomarkers, and those who were tested harbored WT genes for potentially druggable biomarkers.

Treatment approaches documented in this study were in line with current guidelines for Asian countries, where surgery plus SACT and radiotherapy remains the preferred choice for stage IIIA NSCLC.^16^ SACT plus radiotherapy (concurrent chemoradiation) is the preferred choice for stage IIIB NSCLC.

In this study, differences in survival outcomes were observed for the WT subgroup between period 1 (2014-2016) and period 2 (2017-2019). Additionally, there was an increase in the number of patients receiving ICIs from period 1 to period 2. This observation suggests a potential association of ICIs with improved survival outcomes in this patient subgroup. Future studies investigating the association of ICIs with improved patient outcomes, especially among patients with high PDL-1 expression, would be valuable. A significant improvement was also seen in patients with *EGFR*-positive cancer in period 2 compared with period 1. This observation may be attributable to the reimbursement of standardized NGS tests in South Korea for all patients with advanced cancer from 2017 onward, facilitating the early identification of actionable sequence variations and initiation of targeted therapies associated with improved outcomes. On the contrary, no significant difference in survival outcomes was observed between periods among patients with SQ cancer, likely attributable to the absence of novel therapies and limited patients with driver sequence variations receiving targeted therapy.

### Strengths and Limitations

This study is subject to several limitations. Our data sources may not provide a comprehensive picture of patient medical history given that they did not capture encounters outside the 3 hospitals. Because this is a retrospective study, variables of interest missing from the original electronic health records, including treatment failure status, second biopsy molecular results for acquired resistance exploration, and reasons for treatment discontinuation, may affect generalizability. Despite these limitations, we have enhanced the data where possible by abstraction of clinical notes and unstructured data. This study has notable strengths, including analysis of one of the largest cohorts of patients with NSCLC in South Korea. The study included a large population based on broad selection criteria. Thus, these results may reflect well the population of South Korean patients with NSCLC being treated in clinical settings.

## Conclusion

This cohort study of data pooled from multiple clinical data warehouses to produce a large data set has provided detailed insights into the clinical practice of NSCLC management in South Korea, which would not have been possible through single-center studies alone. This emphasizes the value of this landmark multicenter study and common data model approach. Future analysis of EXPLORE-LC datasets, including temporal stratifications, outcomes, and subpopulation analyses, may be done to further assess the association of new therapies with clinical outcomes in the treatment landscape. Moreover, this study highlights the benefits associated with a national-level reimbursement of NGS, which is potentially associated with improved patient outcomes.

## Notes

Supplement 1. **eFigure 1.** Biomarker Results by Druggable Variant Type for Patients With Squamous Cancer **eFigure 2.** Programmed Cell Death Ligand 1 Expressor Levels for Patients With Nonsquamous Cancer and Without Molecular Test Performed **eFigure 3.** Initial Treatment of Stage IIIA and Stage IIIB/C by Year of Diagnosis **eFigure 4.** Sankey Diagram for Treatment Sequence of Patients With Nonsquamous IIIB-IV *EGFR*-Positive Cancer Diagnosed in 2014-2016 and 2017-2019 Treated With Systemic Anticancer Therapy **eFigure 5.** Sankey Diagram for Treatment Sequence of Patients With Nonsquamous IIIB-IV *ALK*-Positive Cancer Diagnosed in 2014-2016 and 2017-2019 Treated With Systemic Anticancer Therapy **eFigure 6.** Sankey Diagram for Treatment Sequence of Patients With Nonsquamous IIIB-IV *EGFR* and *ALK* Wild Type Cancer Diagnosed in 2014-2016 and 2017-2019 Treated With Systemic Anticancer Therapy **eFigure 7.** Sankey Diagram for Treatment Sequence of Patients With Squamous IIIB-IV *EGFR* and *ALK* Wild Type Cancer Diagnosed in 2014-2016 and 2017-2019 Treated With Systemic Anticancer Therapy **eFigure 8.** Overall Survival From Initial Diagnosis by Initial Treatment Received in Stage IIIA and Stage IIIB/C **eTable 1.** Coding Definitions Used to Identify Eligibility Criteria **eTable 2.** Biomarker Testing Results by Histology and Programmed Cell Death Ligand 1 Expression Level **eTable 3.** Survival Outcome From Initial Diagnosis by Driver Mutation and Clinical Stage

Supplement 2.
Data Sharing Statement
