# Supplementary material for: Epidemiology and Outcomes of Non–Small Cell Lung Cancer in South Korea
Source: JAMA Netw Open. 2024 Feb 9;7(2):e2355331. doi: 10.1001/jamanetworkopen.2023.55331 (PMC10858405; doi:10.1001/jamanetworkopen.2023.55331)
Supplement: Supplement 1. — eFigure 1. Biomarker Results by Druggable Variant Type for Patients With Squamous Cancer eFigure 2. Programmed Cell Death Ligand 1 Expressor Levels for Patients With Nonsquamous Cancer and Without Molecular Test Performed eFigure 3. Initial Treatment of Stage IIIA and Stage IIIB/C by Year of Diagnosis eFigure 4. Sankey Diagram for Treatment Sequence of Patients With Nonsquamous IIIB-IV EGFR-Positive Cancer Diagnosed in 2014-2016 and 2017-2019 Treated With Systemic Anticancer Therapy eFigure 5. Sankey Diagram for Treatment Sequence of Patients With Nonsquamous IIIB-IV ALK-Positive Cancer Diagnosed in 2014-2016 and 2017-2019 Treated With Systemic Anticancer Therapy eFigure 6. Sankey Diagram for Treatment Sequence of Patients With Nonsquamous IIIB-IV EGFR and ALK Wild Type Cancer Diagnosed in 2014-2016 and 2017-2019 Treated With Systemic Anticancer Therapy eFigure 7. Sankey Diagram for Treatment Sequence of Patients With Squamous IIIB-IV EGFR and ALK Wild Type Cancer Diagnosed in 2014-2016 and 2017-2019 Treated With Systemic Anticancer Therapy eFigure 8. Overall Survival From Initial Diagnosis by Initial Treatment Received in Stage IIIA and Stage IIIB/C eTable 1. Coding Definitions Used to Identify Eligibility Criteria eTable 2. Biomarker Testing Results by Histology and Programmed Cell Death Ligand 1 Expression Level eTable 3. Survival Outcome From Initial Diagnosis by Driver Mutation and Clinical Stage [file jamanetwopen-e2355331-s001.pdf]

Supplemental Online Content

Jung HA, Lee DH, Lim SM, et al. Epidemiology and outcomes of non–small cell lung cancer in South Korea. *JAMA Netw Open*. 2024;7(2):e2355331.
doi:10.1001/jamanetworkopen.2023.55331

eFigure 1. Biomarker Results by Druggable Variant Type for Patients With Squamous Cancer

eFigure 2. Programmed Cell Death Ligand 1 Expressor Levels for Patients With Nonsquamous Cancer and Without Molecular Test Performed

eFigure 3. Initial Treatment of Stage IIIA and Stage IIIB/C by Year of Diagnosis

eFigure 4. Sankey Diagram for Treatment Sequence of Patients With Nonsquamous IIIB-IV *EGFR*-Positive Cancer Diagnosed in 2014-2016 and 2017-2019 Treated With Systemic Anticancer Therapy

eFigure 5. Sankey Diagram for Treatment Sequence of Patients With Nonsquamous IIIB-IV *ALK*-Positive Cancer Diagnosed in 2014-2016 and 2017-2019 Treated With Systemic Anticancer Therapy

eFigure 6. Sankey Diagram for Treatment Sequence of Patients With Nonsquamous IIIB-IV *EGFR* and *ALK* Wild Type Cancer Diagnosed in 2014-2016 and 2017-2019 Treated With Systemic Anticancer Therapy

eFigure 7. Sankey Diagram for Treatment Sequence of Patients With Squamous IIIB-IV *EGFR* and *ALK* Wild Type Cancer Diagnosed in 2014-2016 and 2017-2019 Treated With Systemic Anticancer Therapy

eFigure 8. Overall Survival From Initial Diagnosis by Initial Treatment Received in Stage IIIA and Stage IIIB/C

eTable 1. Coding Definitions Used to Identify Eligibility Criteria

eTable 2. Biomarker Testing Results by Histology and Programmed Cell Death Ligand 1 Expression Level

eTable 3. Survival Outcome From Initial Diagnosis by Driver Mutation and Clinical Stage

This supplemental material has been provided by the authors to give readers additional information about their work.

© 2024 Jung HA et al. *JAMA Network Open.*

eFigure 1. Biomarker results by druggable mutation type for patients with squamous (SQ) non-small cell lung cancer (NSCLC)

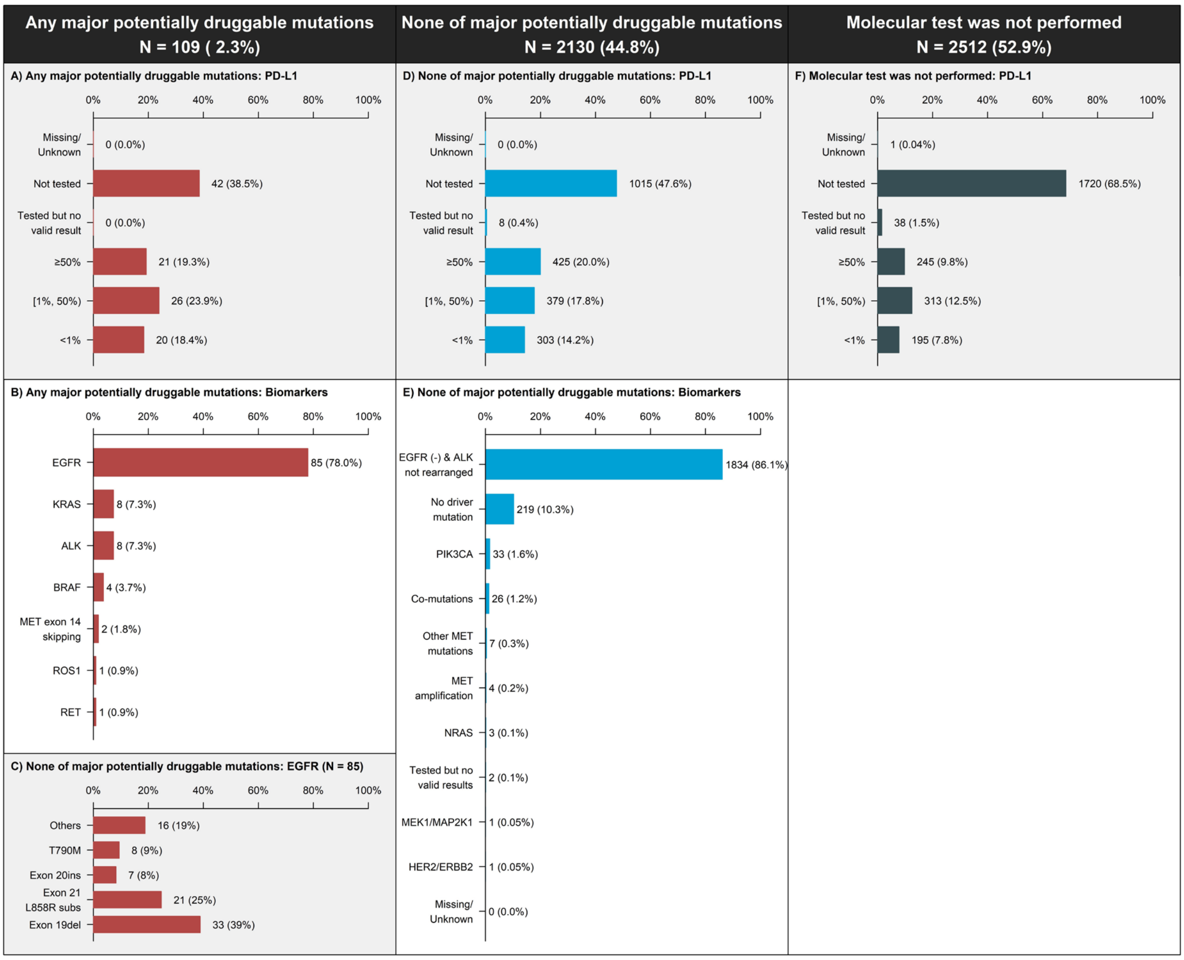

© 2024 Jung HA et al. *JAMA Network Open.*

eFigure 2. PD-L1 expressor levels for patients with non-squamous (NSQ) non-small cell lung cancer (NSCLC) and without molecular test performed

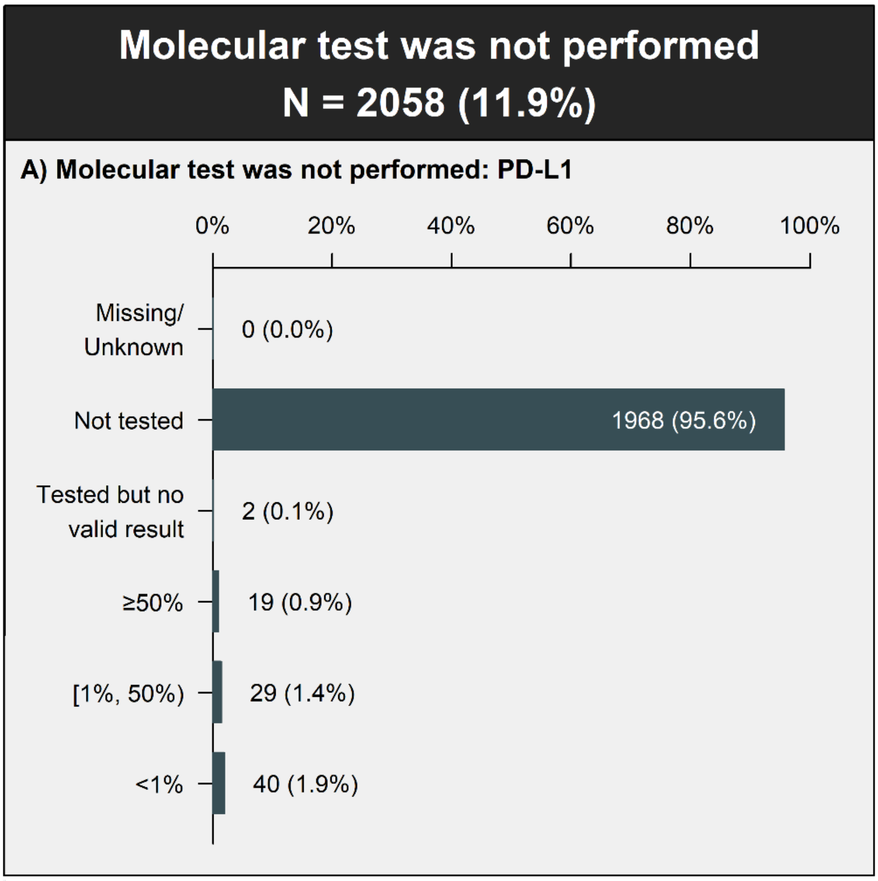

© 2024 Jung HA et al. *JAMA Network Open.*

eFigure 3. Initial treatment of stage IIIA and stage IIIB/C by year of diagnosis

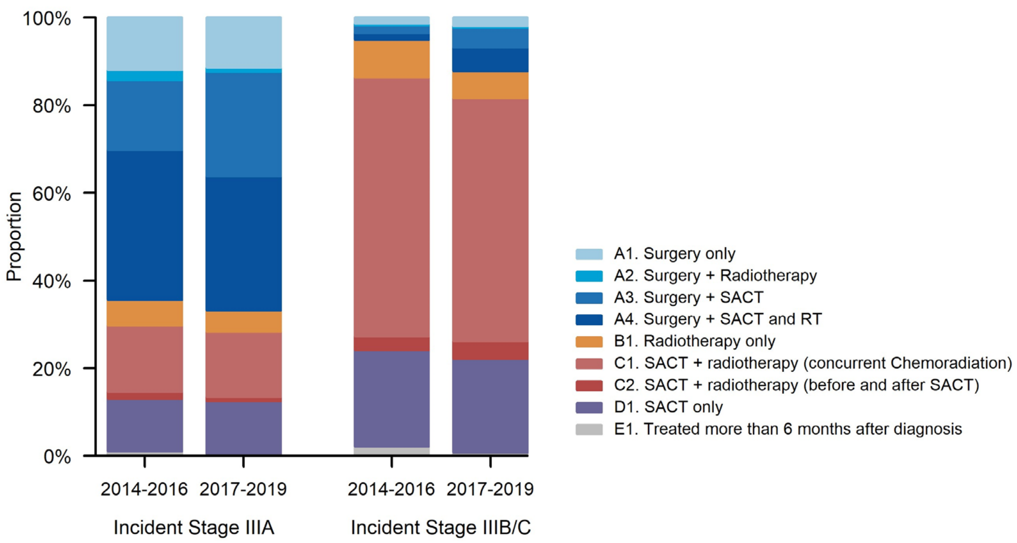

© 2024 Jung HA et al. *JAMA Network Open.*

eFigure 4. Sankey diagram for treatment sequence of SACT-treated NSQ IIIB-IV EGFR positive patients diagnosed in (A) 2014-2016 and (B) 2017-2019

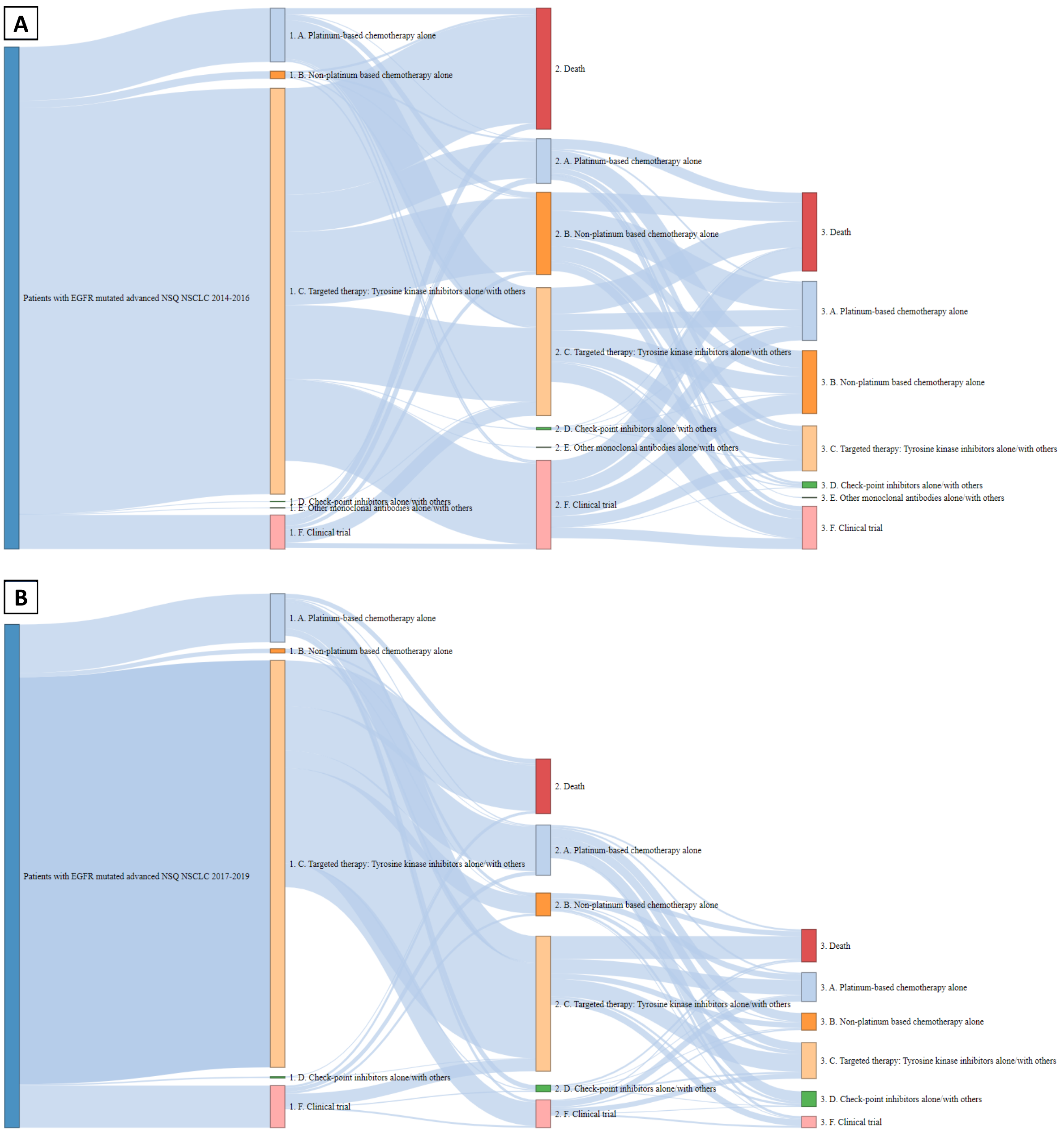

© 2024 Jung HA et al. *JAMA Network Open.*

eFigure 5. Sankey diagram for treatment sequence of SACT-treated NSQ IIIB-IV ALK positive patients diagnosed in (A) 2014-2016 and (B) 2017-2019

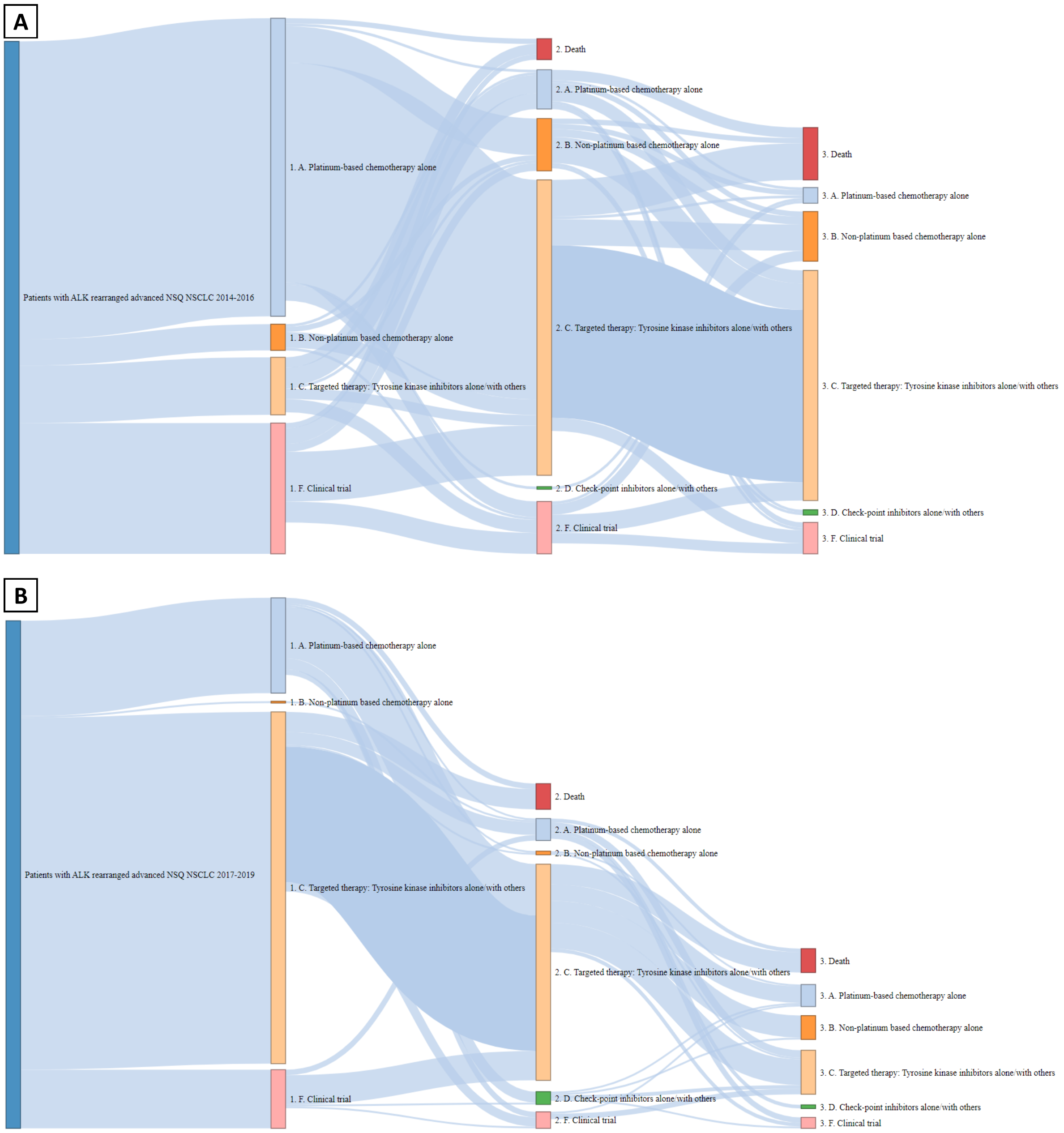

© 2024 Jung HA et al. *JAMA Network Open.*

eFigure 6. Sankey diagram for treatment sequence of SACT-treated NSQ IIIB-IV EGFR and ALK wildtype patients diagnosed in (A) 2014-2016 and (B) 2017-2019

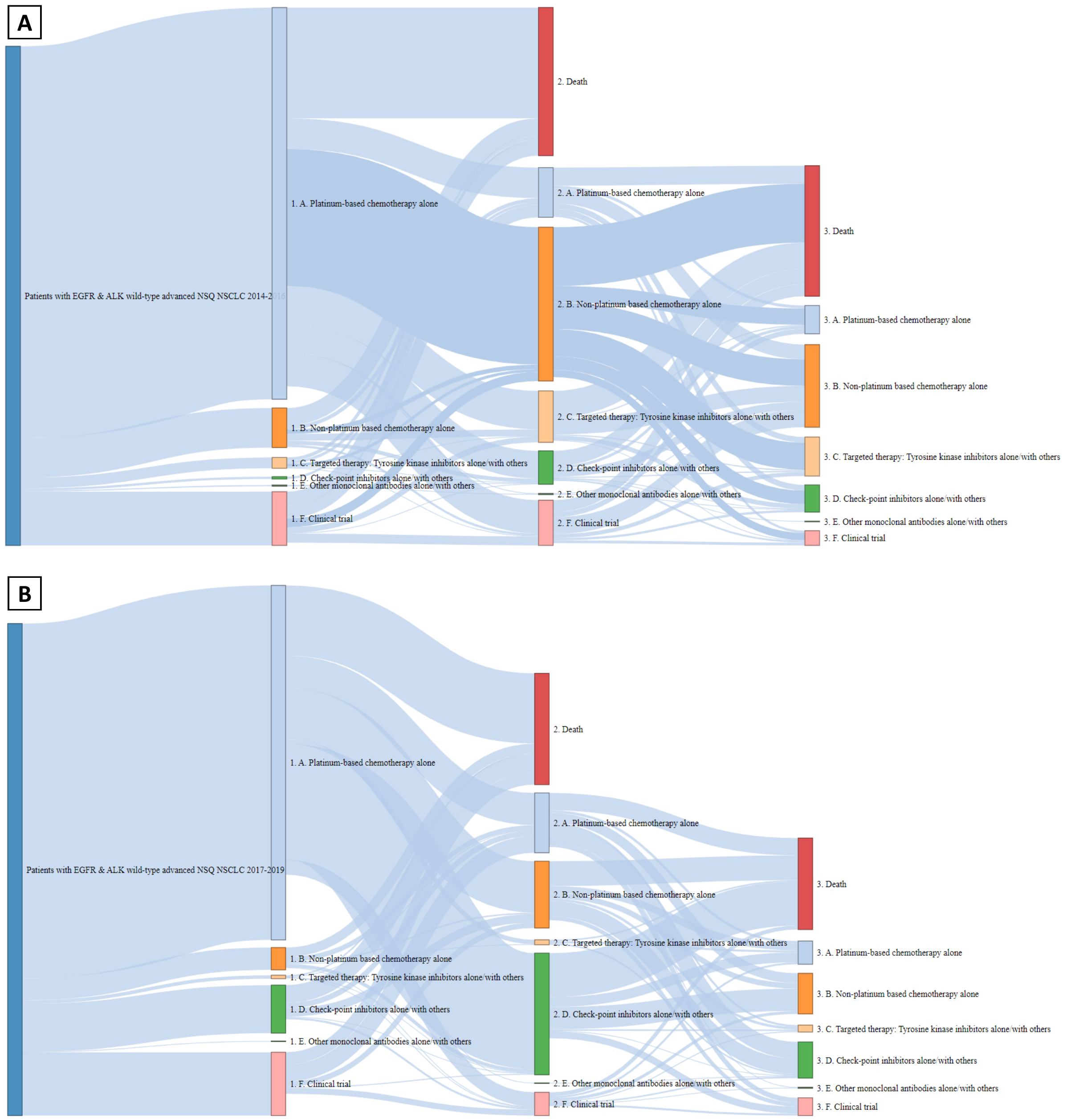

© 2024 Jung HA et al. *JAMA Network Open.*

eFigure 7. Sankey diagram for treatment sequence of SACT-treated SQ IIIB-IV EGFR and ALK wildtype patients diagnosed in (A) 2014-2016 and (B) 2017-2019

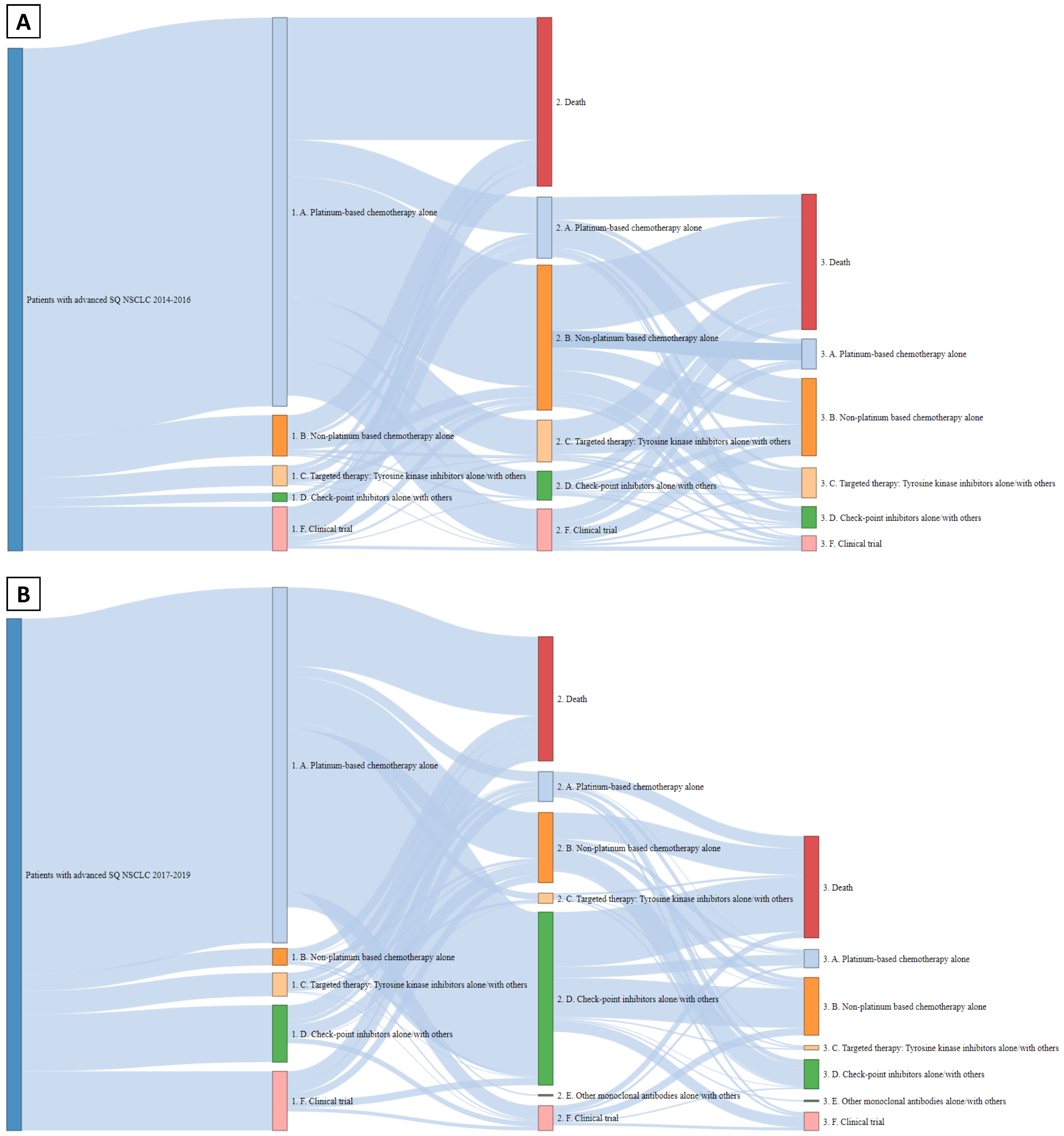

© 2024 Jung HA et al. *JAMA Network Open.*

eFigure 8. Overall survival from initial diagnosis by initial treatment received in (A) stage IIIA and (B) stage IIIB/C.

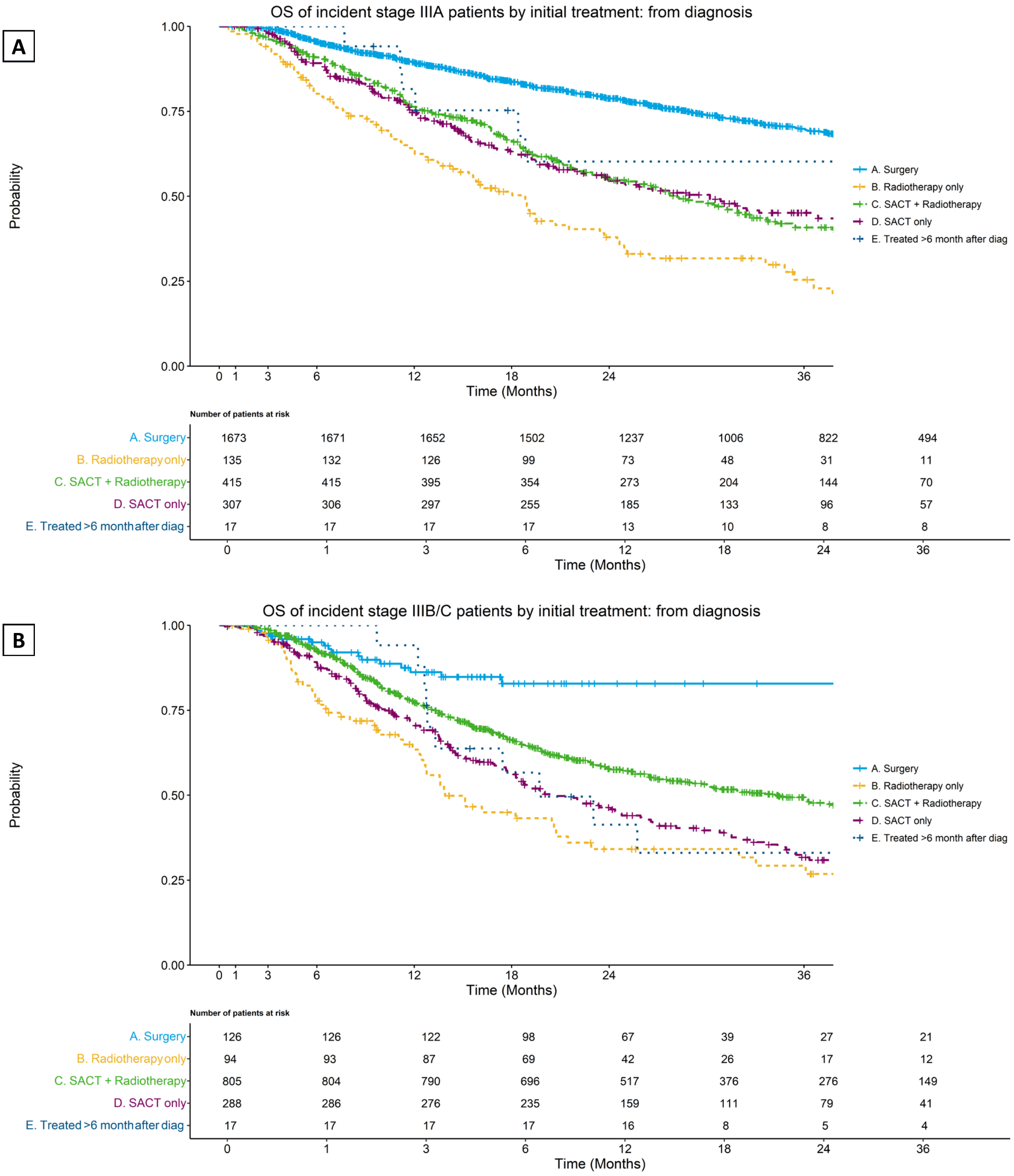

© 2024 Jung HA et al. *JAMA Network Open.*

eTable 1. Coding definitions used to identify eligibility criteria

**Inclusion criteria for NSCLC**	
**NSCLC histopathological type**	**<ICD-O-3>**<br>**Adenocarcinoma (non-squamous NSCLC)**<br>Adenocarcinoma UNS 81403<br>Enteric adenocarcinoma 81443<br>Solid adenocarcinoma with mucin production 82303<br>MANEC mixed adenoneuroendocrine carcinoma 82443<br>Atypical adenomatous hyperplasia 82500<br>Adenocarcinoma in situ, nonmucinous 82502<br>Adenocarcinoma, BAC, bronchiolar carcinoma, (incl pathologic in situ-variant) 82503<br>Bronchio-alveolar carcinoma 82523<br>Adenocarcinoma in situ, mucinous 82532<br>Adenocarcinoma, mucinous BAC 82533<br>Bronchio-alveaolar carcinoma, mixed mucinous and non-mucinous 82543<br>Adenocarcinoma, mixed with other types of carcinoma incl. squamous cell and small-cell carcinoma 82553<br>Minimally invasive adenocarcinoma, nonmucinous 82563<br>Minimally invasive adenocarcinoma, mucinous 82573<br>Papillary adenocarcinoma, NOS 82603<br>Micropapillary adenocarcinoma 82653<br>Clear cell adenocarcinoma 83103<br>Fetal adenocarcinoma 83333<br>Mucinous cystadenocarcinoma 84703<br>Mucinous adenocarcinoma 84803<br>Signet ring cell carcinoma 84903<br>Acinar cell carcinoma 85503<br>Acinar adenocarcinoma 85513<br><br>**Squamous-cell carcinoma**<br>Papillary squamous cell carcinoma 80523<br>Squamous cell carcinoma in situ 80702<br>Squamous cell carcinoma 80703<br>Keratinizing squamous cell carcinoma 80713<br>Non-keratinizing squamous cell carcinoma 80723<br>Squamous cell carcinoma, small cell nonkeratinizing 80733<br>Basaloid squamous cell carcinoma 80833<br>Squamous cell carcinoma, clear cell type 80843<br><br>**NSCLC NOS**<br>Carcinoma, NOS 80103<br>Carcinoma, undifferentiated NOS 80203<br>Carcinoma, anaplastic NOS 80213<br>Carcinoma, non-small cell unspecified 80463<br><br>**Other miscellaneous NSCLC (Other specified NSCLC carcinoma)**<br>Large-cell carcinoma, unspecified 80123<br>Large cell neuroendocrine carcinoma 80133<br>Large cell carcinoma with rhabdoid phenotype 80143<br>Sarcomatoid carcinoma, pleomorphic 80223<br>NUT carcinoma 80233<br>Spindle cell and giant cell carcinoma 80303<br>Giant cell carcinoma 80313<br>Spindle cell carcinoma, NOS 80323

© 2024 Jung HA et al. *JAMA Network Open.*

	Pseudosarcomatous carcinoma 80333<br>Basaloid carcinoma 81233<br>Neuroendocrine carcinoma, NOS 82463<br>Adenocystic carcinoma 82003<br>Mucoepidermoid carcinoma 84303<br>Adenosquamous carcinoma 85603<br>Epithelial-myoepithelial carcinoma 85623<br>Blastoma, pulmonary (pneumoblastoma) 89723<br>Carcinosarkoma, NOS 89803<br>Myoepithelial carcinoma 89823
**Exclusion criteria for previous or concurrent primary cancer, except NSCLC**	
**Previous or concurrent primary cancer, except NSCLC**	**<ICD-10>**<br>C00.x -97.x: Malignant neoplasms (except C34.x)<br>D00.x -09.x: In-situ neoplasms<br>D10.x -36.x: Benign neoplasms<br>D37-48.x: Neoplasms of uncertain or unknown behavior

Abbreviation: BAC, bronchiolo-alveolar; NOS, not otherwise specified; NSCLC, non-small cell lung carcinoma, UNS: Unspecified.

© 2024 Jung HA et al. *JAMA Network Open.*

eTable 2. Biomarker testing results by histology and PD-L1 expression

	Non-squamous carcinoma							Squamous carcinoma						
	**All (n=17350)**	**Not tested (n=10405)**	**Tested / not valid[a] (n=142)**	**PD-L1 <1% (n=3386)**	**PD-L1 ≥ 1 % and < 50% (n=1841)**	**PD-L1 ≥ 50% (n=1570)**	**Miss / Unk[b] (n=6)**	**All (n=4751)**	**Not tested (n=2777)**	**Tested / not valid[a] (n=46)**	**PD-L1 <1% (n=518)**	**PD-L1 ≥ 1 % and < 50% (n=718)**	**PD-L1 ≥ 50% (n=691)**	**Miss / Unk[b] (n<3)**
	**No. (%)**	**No. (%)**	**No. (%)**	**No. (%)**	**No. (%)**	**No. (%)**	**No. (%)**	**No. (%)**	**No. (%)**	**No. (%)**	**No. (%)**	**No. (%)**	**No. (%)**	**No. (%)**
Not tested	2058 (11.9)	1968 (18.9)	< 3 (*)	40 (1.2)	29 (1.6)	19 (1.2)	0 (0.0)	2512 (52.9)	1720 (61.9)	38 (82.6)	195 (37.6)	313 (43.6)	245 (35.5)	< 3 (*)
Tested/not valid[a]	5 (0.0)[c]	5 (0.1)	0 (0.0)	0 (0.0)	0 (0.0)	0 (0.0)	0 (0.0)	< 3 (*)	< 3 (*)	0 (0.0)	0 (0.0)	0 (0.0)	0 (0.0)	0 (0.0)
Co-mutations	391 (2.3)	126 (1.2)	4 (2.8)	100 (3.0)	68 (3.7)	93 (5.9)	0 (0.0)	26 (0.6)	4 (0.1)	0 (0.0)	6 (1.2)	7 (1.0)	9 (1.3)	0 (0.0)
**Single mutation (Non-squamous carcinoma: 8332, 48.0%; Squamous carcinoma: 158, 3.3%)**														
ALK	914 (5.3)	526 (5.2)	8 (5.6)	116 (3.4)	119 (6.5)	145 (9.2)	0 (0.0)	8 (0.2)	3 (0.1)	0 (0.0)	4 (0.8)	< 3 (*)	0 (0.0)	0 (0.0)
BRAF	32 (0.2)	9 (0.1)	0 (0.0)	8 (0.2)	6 (0.3)	9 (0.6)	0 (0.0)	4 (0.1)	0 (0.0)	0 (0.0)	< 3 (*)	< 3 (*)	< 3 (*)	0 (0.0)
EGFR[d]	6866 (39.6)	3756 (36.1)	43 (30.3)	1777 (52.5)	825 (44.8)	464 (29.6)	< 3 (*)	85 (1.8)	37 (1.3)	0 (0.0)	10 (1.9)	20 (2.8)	18 (2.6)	0 (0.0)
E19del	2999 (17.3)	1684 (16.2)	12 (8.5)	747 (22.1)	358 (19.5)	197 (12.6)	< 3 (*)	33 (0.7)	13 (0.5)	0 (0.0)	6 (1.2)	4 (0.6)	10 (1.5)	0 (0.0)
E20ins	185 (1.1)	71 (0.7)	0 (0.0)	53 (1.6)	37 (2.0)	24 (1.5)	0 (0.0)	7 (0.2)	< 3 (*)	0 (0.0)	< 3 (*)	< 3 (*)	< 3 (*)	0 (0.0)
E21L858Rsub	2591 (14.9)	1449 (13.9)	8 (5.6)	718 (21.2)	262 (14.2)	154 (9.8)	0 (0.0)	21 (0.4)	10 (0.4)	0 (0.0)	< 3 (*)	6 (0.8)	4 (0.6)	0 (0.0)
T790M	556 (3.2)	258 (2.5)	8 (5.6)	147 (4.3)	94 (5.1)	49 (3.1)	0 (0.0)	8 (0.2)	5 (0.2)	0 (0.0)	0 (0.0)	3 (0.4)	0 (0.0)	0 (0.0)
Others	535 (3.1)	294 (2.8)	15 (10.6)	112 (3.3)	74 (4.0)	40 (2.6)	0 (0.0)	16 (0.3)	7 (0.3)	0 (0.0)	< 3 (*)	5 (0.7)	3 (0.4)	0 (0.0)
HER2 or ERBB2	43 (0.3)	6 (0.1)	0 (0.0)	20 (0.6)	11 (0.6)	6 (0.4)	0 (0.0)	< 3 (*)	0 (0.0)	0 (0.0)	0 (0.0)	< 3 (*)	0 (0.0)	0 (0.0)
KRAS	239 (1.4)	92 (0.9)	0 (0.0)	44 (1.3)	53 (2.9)	50 (3.2)	0 (0.0)	8 (0.2)	< 3 (*)	0 (0.0)	4 (0.8)	< 3 (*)	< 3 (*)	0 (0.0)
MEK1 / MAP2K1	9 (0.1)	3 (0.0)[c]	0 (0.0)	4 (0.1)	< 3 (*)	< 3 (*)	0 (0.0)	< 3 (*)	0 (0.0)	0 (0.0)	< 3 (*)	0 (0.0)	0 (0.0)	0 (0.0)
MET e14 skip[e]	14 (0.1)	< 3 (*)	0 (0.0)	4 (0.1)	3 (0.2)	5 (0.3)	0 (0.0)	< 3 (*)	0 (0.0)	0 (0.0)	0 (0.0)	< 3 (*)	< 3 (*)	0 (0.0)
MET amp[f]	26 (0.2)	3 (0.0)[c]	0 (0.0)	3 (0.1)	7 (0.4)	13 (0.8)	0 (0.0)	4 (0.1)	< 3 (*)	0 (0.0)	< 3 (*)	< 3 (*)	< 3 (*)	0 (0.0)
Other MET	19 (0.1)	< 3 (*)	0 (0.0)	6 (0.2)	4 (0.2)	7 (0.5)	0 (0.0)	7 (0.2)	< 3 (*)	0 (0.0)	< 3 (*)	3 (0.4)	< 3 (*)	0 (0.0)
NRAS	< 3 (*)	0 (0.0)	0 (0.0)	0 (0.0)	< 3 (*)	< 3 (*)	0 (0.0)	3 (0.1)	< 3 (*)	0 (0.0)	< 3 (*)	0 (0.0)	< 3 (*)	0 (0.0)
PIK3CA	11 (0.1)	< 3 (*)	0 (0.0)	4 (0.1)	< 3 (*)	5 (0.3)	0 (0.0)	33 (0.7)	4 (0.1)	0 (0.0)	8 (1.5)	13 (1.8)	8 (1.2)	0 (0.0)
RET	59 (0.3)	13 (0.1)	0 (0.0)	14 (0.4)	15 (0.8)	16 (1.0)	< 3 (*)	< 3 (*)	< 3 (*)	0 (0.0)	0 (0.0)	0 (0.0)	0 (0.0)	0 (0.0)

© 2024 Jung HA et al. *JAMA Network Open.*

ROS1	98 (0.6)	20 (0.2)	3 (2.1)	22 (0.7)	24 (1.3)	29 (1.9)	0 (0.0)	< 3 (*)	0 (0.0)	0 (0.0)	0 (0.0)	< 3 (*)	0 (0.0)	0 (0.0)
EGFR(-)&ALK(-)	5304 (30.6)	2700 (26.0)	80 (56.3)	1189 (35.1)	648 (35.2)	683 (43.5)	4 (66.7)	1834 (38.6)	879 (31.7)	6 (13.0)	264 (51.0)	313 (43.6)	372 (53.8)	0 (0.0)
No driver mutation	1254 (7.2)	1168 (11.2)	< 3 (*)	35 (1.0)	26 (1.4)	23 (1.5)	0 (0.0)	219 (4.6)	122 (4.4)	< 3 (*)	21 (4.1)	41 (5.7)	33 (4.8)	0 (0.0)
Miss / Unk[b]	6 (0.0)[c]	5 (0.1)	0 (0.0)	0 (0.0)	0 (0.0)	< 3 (*)	0 (0.0)	0 (0.0)	0 (0.0)	0 (0.0)	0 (0.0)	0 (0.0)	0 (0.0)	0 (0.0)
**PD-L1 expression**														
Not tested	10405 (60.0)	10405 (100.0)	0 (0.0)	0 (0.0)	0 (0.0)	0 (0.0)	0 (0.0)	2777 (58.5)	2777 (100.0)	0 (0.0)	0 (0.0)	0 (0.0)	0 (0.0)	0 (0.0)
Tested/not valid[a]	142 (0.8)	0 (0.0)	142 (100.0)	0 (0.0)	0 (0.0)	0 (0.0)	0 (0.0)	46 (1.0)	0 (0.0)	46 (100.0)	0 (0.0)	0 (0.0)	0 (0.0)	0 (0.0)
PD-L1 <1%	3386 (19.5)	0 (0.0)	0 (0.0)	3386 (100.0)	0 (0.0)	0 (0.0)	0 (0.0)	518 (10.9)	0 (0.0)	0 (0.0)	518 (100.0)	0 (0.0)	0 (0.0)	0 (0.0)
PD-L1 ⩾ 1&<50%	1841 (10.6)	0 (0.0)	0 (0.0)	0 (0.0)	1841 (100.0)	0 (0.0)	0 (0.0)	718 (15.1)	0 (0.0)	0 (0.0)	0 (0.0)	718 (100.0)	0 (0.0)	0 (0.0)
PD-L1 ⩾50%	1570 (9.1)	0 (0.0)	0 (0.0)	0 (0.0)	0 (0.0)	1570 (100.0)	0 (0.0)	691 (14.5)	0 (0.0)	0 (0.0)	0 (0.0)	0 (0.0)	691 (100.0)	0 (0.0)
Miss / Unk[b]	6 (0.0)[c]	0 (0.0)	0 (0.0)	0 (0.0)	0 (0.0)	0 (0.0)	6 (100.0)	< 3 (*)	0 (0.0)	0 (0.0)	0 (0.0)	0 (0.0)	0 (0.0)	< 3 (*)

Abbreviations: ALK, Anaplastic lymphoma kinase; BRAF, B-Raf proto-oncogene; ECOG, Eastern Cooperative Oncology Group; EGFR, Epidermal growth factor receptor; ERBB2, erythroblastic oncogene B; HER2, human epidermal growth factor receptor 2; KRAS, kirsten rat sarcoma virus; MET, mesenchymal epithelial transition; NRAS, neuroblastoma-RAS; PD-L1, programmed death-ligand 1; PIK3CA, phosphatidylinositol-4,5-bisphosphate 3-kinase catalytic subunit alpha; RET, rearranged during transfection; ROS1, c-ros oncogene 1; SD, standard deviation; TNM, Tumor Node Metastasis; WT, wildtype.

[a]Tested but not valid result.

[b]Missing or Unknown result.

[c]0.0% = 0.03%.

[d]E19del, exon 19 deletion; E20ins, exon 20 insertion; E21L858Rsub, exon 21 L858R substitution; T790M, Thr790Met.

[e]exon 14 skipping.

[f]amplification.

© 2024 Jung HA et al. *JAMA Network Open.*

eTable 3. Survival outcome from initial diagnosis by driver mutation and clinical stage

Patient subgroup	Clinical stage	Total					12 Months		24 months		36 months	
		No. of patients at risk (N)	No. of deaths (N)	No. of censored patients (N)	Median (95% CI) in months	Q1 - Q3 in months	N	Probability [95% CI]	N	Probability [95% CI]	N	Probability [95% CI]
**Non-squamous carcinoma patients**												
EGFR+[a]	I	3137	100	3037	not reached [n.a,n.a]	n.a-n.a	2634	1.00 [0.99,1.00]	1967	0.99 [0.98,0.99]	1332	0.97 [0.96,0.98]
	II	462	46	416	not reached [74.53,n.a]	74.53-n.a	366	0.97 [0.96,0.99]	256	0.94 [0.92,0.97]	158	0.89 [0.85,0.93]
	IIIA	461	76	385	not reached [61.3,n.a]	44.03-n.a	368	0.97 [0.95,0.99]	261	0.92 [0.89,0.95]	162	0.84 [0.80,0.89]
	IIIB/C	173	43	130	59.6 [47.77,n.a]	34.37-n.a	126	0.92 [0.88,0.97]	78	0.82 [0.75,0.89]	40	0.70 [0.61,0.80]
	IV	2633	1171	1462	34.6 [32.23,36.07]	18.23-65.3	1930	0.86 [0.84,0.87]	1112	0.65 [0.63,0.67]	543	0.48 [0.45,0.50]
ALK+[b]	I	231	7	224	not reached [n.a,n.a]	n.a-n.a	201	1.00 [1.00,1.00]	156	1.00 [1.00,1.00]	122	0.99 [0.98,1.00]
	II	67	11	56	not reached [62.63,n.a]	62.63-n.a	53	0.94 [0.88,1.00]	35	0.92 [0.85,0.99]	22	0.80 [0.69,0.93]
	IIIA	103	11	92	not reached [n.a,n.a]	n.a-n.a	89	0.98 [0.95,1.00]	62	0.93 [0.88,0.99]	37	0.86 [0.79,0.95]
	IIIB/C	57	12	45	not reached [39.1,n.a]	25.73-n.a	47	0.94 [0.88,1.00]	27	0.87 [0.77,0.97]	14	0.69 [0.55,0.87]
	IV	456	147	309	56.37 [48,n.a]	24.13-n.a	341	0.88 [0.85,0.91]	218	0.75 [0.71,0.80]	128	0.62 [0.56,0.67]
EGFR WT and ALK WT	I	2020	136	1884	not reached [n.a,n.a]	n.a-n.a	1672	0.99 [0.98,0.99]	1281	0.96 [0.95,0.97]	859	0.93 [0.91,0.94]
	II	527	115	412	not reached [63.37,n.a]	36.63-n.a	410	0.93 [0.90,0.95]	272	0.82 [0.79,0.86]	178	0.76 [0.71,0.80]
	IIIA	549	213	336	38.07 [31.93,58.47]	16.37-n.a	369	0.83 [0.80,0.86]	226	0.65 [0.61,0.70]	123	0.52 [0.47,0.57]
	IIIB/C	311	152	159	23.33 [19.87,35.47]	11.17-n.a	183	0.73 [0.68,0.78]	92	0.49 [0.43,0.55]	55	0.42 [0.36,0.49]

© 2024 Jung HA et al. *JAMA Network Open.*

	IV	1897	1376	521	12.03 [11.37,12.8]	5.87-24.57	788	0.50 [0.48,0.53]	312	0.26 [0.24,0.28]	131	0.16 [0.14,0.18]
**Squamous carcinoma patients**												
Diagnosis year 2014-2016	I	610	175	435	not reached [n.a,n.a]	38.93-n.a	547	0.92 [0.90,0.95]	476	0.84 [0.81,0.87]	412	0.76 [0.73,0.80]
	II	485	213	272	68.63 [51.83,n.a]	19.07-n.a	408	0.86 [0.83,0.90]	324	0.70 [0.66,0.74]	274	0.61 [0.56,0.65]
	IIIA	491	306	185	27.20 [24.1,32.93]	12.03-n.a	365	0.75 [0.71,0.79]	256	0.54 [0.50,0.59]	191	0.42 [0.38,0.47]
	IIIB/C	207	147	60	21.47 [17.73,28.13]	10.63-n.a	146	0.71 [0.65,0.77]	94	0.46 [0.40,0.53]	72	0.36 [0.30,0.43]
	IV	485	439	46	10.23 [9.17,11.33]	5.7-20.3	198	0.41 [0.37,0.46]	104	0.22 [0.18,0.26]	62	0.13 [0.11,0.17]
Diagnosis year 2017-2019	I	555	40	515	not reached [n.a,n.a]	n.a-n.a	383	0.96 [0.94,0.98]	204	0.92 [0.89,0.95]	37	0.88 [0.83,0.92]
	II	493	80	413	not reached [n.a,n.a]	29.5-n.a	312	0.91 [0.88,0.93]	154	0.81 [0.77,0.85]	25	0.71 [0.65,0.78]
	IIIA	496	128	368	not reached [37.5,n.a]	16.17-n.a	275	0.78 [0.74,0.82]	117	0.65 [0.60,0.70]	22	0.62 [0.56,0.68]
	IIIB/C	336	124	212	20.60 [17.63,n.a]	10.07-n.a	158	0.71 [0.66,0.77]	42	0.47 [0.40,0.55]	6	0.41 [0.33,0.51]
	IV	593	338	255	11.27 [10.23,12.4]	5.53-25.43	185	0.47 [0.43,0.52]	48	0.27 [0.22,0.32]	0	0.20 [0.15,0.26]

Abbreviations: ALK, Anaplastic lymphoma kinase; EGFR, Epidermal growth factor receptor; n.a, not applicable; WT, wildtype.

[a]EGFR mutations, defined as all EGFR-activating mutations includes but not limited to exon 19 deletion, exon 21 L858R substitution, and exon 20 insertion.

[b]ALK mutations, also known as ALK rearrangements or translocation.

© 2024 Jung HA et al. *JAMA Network Open.*
