# Supplementary material for: Epidemiology and Outcomes of Non–Small Cell Lung Cancer in South Korea
Source: JAMA Netw Open. 2024 Feb 9;7(2):e2355331. doi: 10.1001/jamanetworkopen.2023.55331 (PMC10858405; doi:10.1001/jamanetworkopen.2023.55331)
Supplement: Supplement 2. — Data Sharing Statement [file jamanetwopen-e2355331-s002.pdf]

Data Sharing Statement

Jung. Epidemiology and Outcomes of Non–Small Cell Lung Cancer in South Korea. *JAMA Network Open*. Published online February 9, 2024. doi:10.1001/jamanetworkopen.2023.55331

Data
Data available: No
